# Corrigendum to Angiopoietin-2 exacerbates cardiac hypoxia and inflammation after myocardial infarction

**DOI:** 10.1172/JCI205881

**Published:** 2026-04-01

**Authors:** Seung-Jun Lee, Choong-kun Lee, Seok Kang, Intae Park, Yoo Hyung Kim, Seo Ki Kim, Seon Pyo Hong, Hosung Bae, Yulong He, Yoshiaki Kubota, Gou Young Koh

Original citation: *J Clin Invest*. 2018;128(11):5018–5033. https://doi.org/10.1172/JCI99659

Citation for this corrigendum: *J Clin Invest*. 2026;136(7):e205881. https://doi.org/10.1172/JCI205881

The authors recently became aware of the following errors in the original manuscript: In Figure 7K, the ATN-161 image was incorrect and was derived from the same sample as the Figure 7H WT image; in Supplemental Figure 6B, the WT TER119/NG2/CD31 image was incorrect and was derived from the same sample as the Supplemental 6B *Tie2^iΔEC^* TER119/NG2/CD31 image; in Supplemental Figure 8B, the MI 3d images were incorrect and were derived from the same samples as Figure 6E *Angpt2^iΔEC^*; in Supplemental Figure 11A, the Sham ZO1/CD31 image was incorrect and was derived from the same sample as Figure 9D Sham FITC-Lectin/CD31; and in Supplemental Figure 13A, the I/R 7d Angpt2/CD31 image was incorrect and was derived from the same sample as shown in Figure 3F. The authors confirmed that the quantitative data accompanying these errors were obtained independently and were unaffected by the corrections. The legend for Supplemental Figure 8B was also updated for clarity and accuracy. The corrected figures, based on the original source data, are provided below and in the updated supplemental materials. The HTML and PDF versions of the paper have been updated.

The authors regret the errors.

## Supplementary Material

Supplemental data

## Figures and Tables

**Figure 7:**
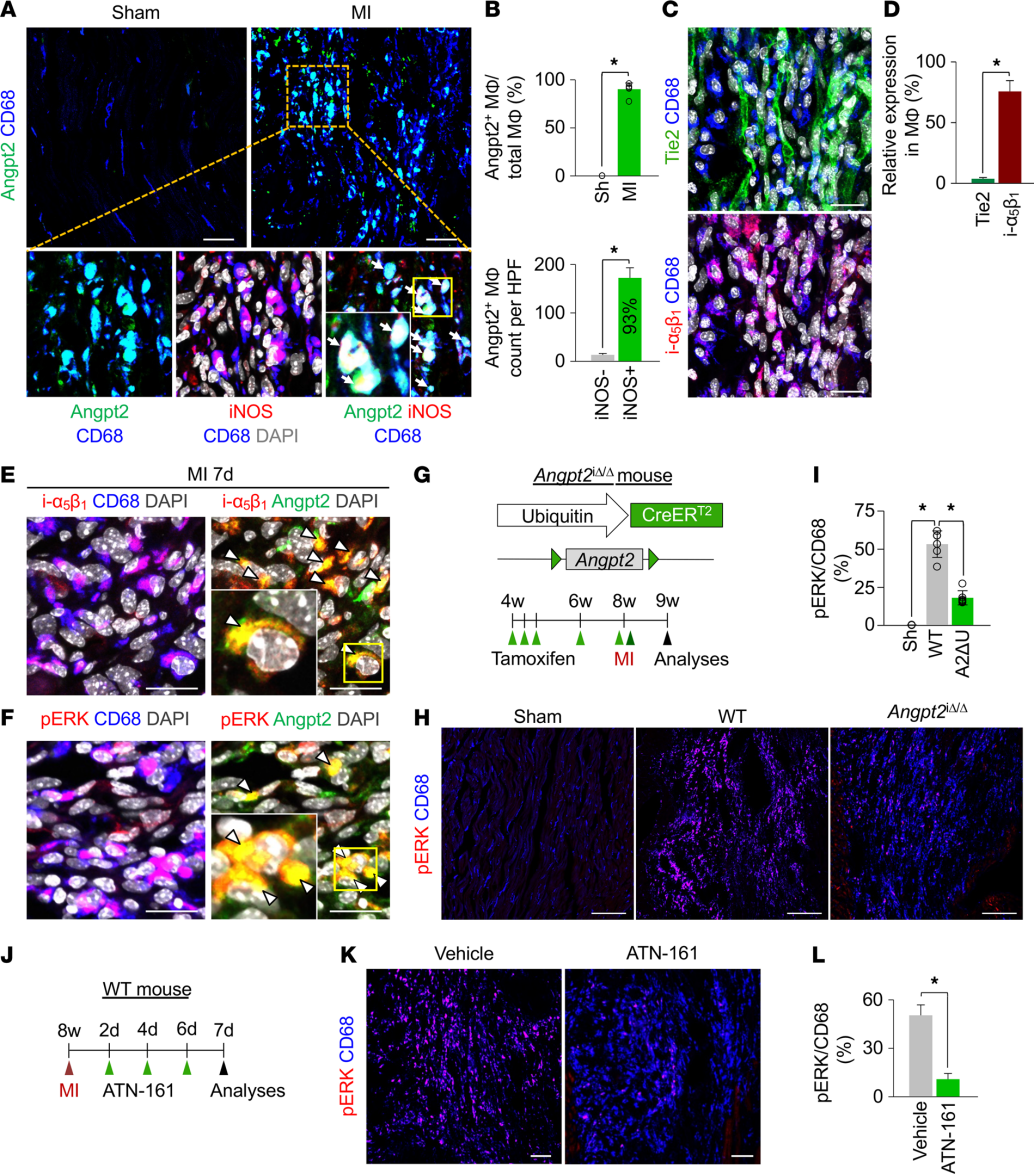
Angpt2/integrin α_5_β_1_ signaling is positively associated with pERK expression in macrophages in ischemic heart.

## Version 1 04/01/2026

Electronic publication

