# Supplementary material for: Corrigendum to Angiopoietin-2 exacerbates cardiac hypoxia and inflammation after myocardial infarction
Source: J Clin Invest. 2026 Apr 1;136(7):e205881. doi: 10.1172/JCI205881 (PMC13038188; doi:10.1172/JCI205881)
Supplement: Supplemental data [file jci-136-205881-s182.pdf]

Supplemental Materials for:

Angiopoietin-2 Exacerbates Cardiac Hypoxia and Inflammation after Myocardial Infarction

Seung-Jun Lee, Choong-kun Lee, Seok Kang, Intae Park, Yoo Hyung Kim, Seo Ki Kim, Seon Pyo Hong, Hosung Bae, Yulong He, Yoshiaki Kubota, Gou Young Koh*

<u>This material includes:</u>

Supplemental Methods and related References

Supplementary Table

Supplemental Figures and Legends

Supplemental Methods

Histological analyses

At indicated time points, heart was harvested after perfusion-fixation and fixed in 4% paraformaldehyde (PFA) in PBS at 4°C for 6 hours. For measurement of the infarct size and fibrosis at 3 weeks after MI, tissues were processed using standard procedures, embedded in paraffin, and cut into 3 µm sections at the level of papillary muscle, and followed by hematoxylin and eosin (H&E) staining or Masson's trichrome staining. The infarct size was calculated as the infarct circumference divided by total left ventricular circumference (1). The wall thickness of the scar area was measured at the thinnest part. To evaluated the infarct size at 3 days after MI, the harvested hearts were frozen at −20°C for 1 hour, cut transversely in 1 mm thickness using a Mouse Heart Slicer Matrix (ROBOZ Surgical Instruments), and stained with 1% triphenyltetrazolium chloride (TTC) in PBS (pH 7.4) for 20 minutes at 37°C. Infarct area and wall thickness visualized by H&E, Masson's trichrome, and TTC staining was analyzed using ImageJ software (http://rsb.info.nih.gov/ij). For immunofluorescence staining, samples were further processed by dehydration in 15% and 30% sucrose, consecutively, and embedded in tissue freezing medium (Leica). Frozen blocks were cut into 25 µm sections. Samples were blocked with 5% goat or donkey serum in PBST (0.3% Triton X-100 in PBS) and then incubated in blocking solution with one or more of the following antibodies at 4°C overnight: anti-CD31 (hamster monoclonal, clone 2H8, Millipore, MAB1398Z); anti-Angpt2 (human monoclonal, clone 4H10) (2); anti-NG2 (rabbit monoclonal, clone 132.39, Millipore, MAB5384); anti-GFP (rabbit polyclonal, Millipore, AB3080); anti-

CD117 (goat polyclonal, R&D, AF1356); anti-FOXO1 (rabbit monoclonal, clone C29H4, Cell signaling, 2880); anti-Tie1 (goat polyclonal, R&D, AF619); anti-TER119 (rat monoclonal, clone TER-119, BD Pharmingen, 561033); anti-GLUT1 (rabbit polyclonal, Millipore, 07-1401); anti-E-selectin (rat monoclonal, clone 10E9.6, BD Pharmingen, 550290); anti-L-selectin (rat monoclonal, clone MEL-14, BD Pharmingen, 553149); anti-VCAM-1 (rat monoclonal, clone M/K-2, Abcam, ab19569); anti-ICAM-1 (hamster monoclonal, clone 3E2, BD Pharmingen, 550287); anti-Gr-1 (rat monoclonal, clone RB6-8C5, eBioscience, 13-5931-85); anti-NF-κB p65 (rabbit monoclonal, clone D14E12, Cell signaling, 8242); anti-heparanase (rabbit polyclonal, InSight, INS-26-2); anti-heparan sulfate (mouse monoclonal, clone F58-10E4, Amsbio, 370255-1); anti-CD45 (rat monoclonal, clone 30-F11, BD Pharmingen, 550539); anti-iNOS (rabbit polyclonal, Abcam, ab15323); anti-CD68 (rat monoclonal, clone FA-11, Bio-Rad, MCA1957); anti-integrin α5β1 (rat monoclonal, clone BMB5, Millipore, MAB2514); anti-phospho-ERK (Rabbit monoclonal, clone 197G2, Cell signaling, 4377); anti-CD206 (rat monoclonal, C068C2, Biolegend, 141710); anti-MHC class II (rat monoclonal, M5/114.15.2, eBioscience, 47-5321-80); anti-Arginase 1 (mouse monoclonal, clone C-2, Santacruz, sc-166920); anti-CD29 (rat monoclonal, clone 9EG7, BD Pharmingen, 550531); anti-collagen type IV (rabbit polyclonal, Abcam, ab6586); anti-ZO-1 (rabbit polyclonal, Thermo Fisher Scientific, 61-7300); anti-phospho-FAK (rabbit polyclonal, Abcam, ab39967); anti-fibronectin (rabbit polyclonal, Millipore, ab2033). After several washes, the samples were incubated at room temperature (RT) for 2 hours with the following secondary antibodies: FITC-, Cy3-, or Cy5- conjugated anti-hamster IgG, anti-rabbit IgG, anti-rat IgG, anti-goat IgG, and anti-human IgG antibody (Jackson ImmunoResearch). Nuclei were stained with 4′,6-diamidino-2-

phenylindole (DAPI, Invitrogen). Samples were mounted with fluorescent mounting medium (DAKO) and immunofluorescence images were acquired with a Zeiss LSM880 confocal microscope (Carl Zeiss). To detect hypoxic areas of the infarct border, Hypoxyprobe-1^{TM} (60 mg/kg, solid pimonidazole hydrochloride, Natural Pharmacia International) was intraperitoneally injected 60 minutes before perfusion fixation. Hearts were then harvested, sectioned, and stained with FITC-conjugated anti-Hypoxyprobe antibody.

Morphometric analyses

Morphometric analyses of the heart were performed using ImageJ software (http://rsb.info.nih.gov/ij) or ZEN 2012 software (Carl Zeiss). For statistical analysis, three random areas were measured in each side of infarct border and the sum was averaged, unless indicated otherwise. Relative Angpt2 expression on ECs was calculated as Angpt2$^+$ area divided by CD31$^+$ EC area. Angpt2-EGFP expression was measured as EGFP$^+$ area divided by CD31$^+$ EC area per random 1.7 mm^2 areas. Relative Angpt2 expression on the cardiac resident ECs was calculated as a percentage of Angpt2$^+$ length along the CD144-tdTomato$^+$ EC area in 0.17 mm^2 areas. Pericyte coverage was calculated as NG2$^+$ area divided by the total CD31$^+$ EC area. FOXO1$^+$ EC was calculated as % of FOXO1$^+$ length along the CD31$^+$ EC area in 0.17 mm^2 areas. FOXO1$^+$ CM was calculated as FOXO1$^+$ area outside the vessels divided by WGA$^+$ area in 0.17 mm^2 areas. Angpt1-GFP expression was measured as GFP$^+$ area divided α-actinin$^+$ myocardium area per 8.0 mm^2 areas. pTie2$^+$ EC was calculated as % of pTie2$^+$ length along the CD31$^+$ EC area in 0.56 mm^2 areas. Relative Tie1 intensity was calculated as Tie1$^+$ area divided by CD31$^+$ EC area. RBC leakage was measure as TER119$^+$ area

outside the vessels divided by $CD31^+$ EC area. Hypoxic status of infarct border was measured as a % of GLUT1+ area divided by CD31+ EC area in 2.4 mm^2 areas. Adhesion molecule expression was calculated as % of E-selectin$^+$, L-selectin$^+$, VCAM-1$^+$, or ICAM-1$^+$ length along the $CD31^+$ EC area. Neutrophil infiltration was counted as Gr-1$^+$ cells in two 6.3 mm^2 areas of each infarct border. Leukocyte infiltration was calculated as $CD45^+$ area divided by $CD31^+$ EC area. NF-κB p65 and heparanase expression was calculated as % of corresponding fluorescent positive area divided by $CD31^+$ EC area. To define HS into glycocalyx and ECM, we utilized ImageJ software. Among the whole HS components, the portion overlapping with CD31 was defined as HS-eGC, and the other portion was defined as HS-cECM, respectively. Then, relative HS, HS-eGC and HS-cECM density was calculated as % of corresponding fluorescent positive area divided by $CD31^+$ EC area. Relative Angpt2 expression on cardiac macrophages was calculated as $Angpt2^+$ & $CD68^+$ area divided by $CD68^+$ macrophage area. ERK phosphorylation of the macrophages was calculated as $pERK^+$ & $CD68^+$ area divided by $CD68^+$ macrophage area in 0.31 mm^2 areas. Cytokine expression profile of macrophages was calculated as $iNOS^+$, $CD206^+$, MHC II$^+$, or Arg-1$^+$ & $CD68^+$ area divided by $CD68^+$ macrophage area, respectively. Relative integrin β1 activation was calculated as a percentage of $CD29^+$ length along the $CD31^+$ EC area. Collagen type IV$^+$ basement membrane coverage and ZO-1$^+$ tight junction distribution was calculated as % of corresponding fluorescent positive length along the $CD31^+$ EC area in 0.15 mm^2 areas of each infarct border. Measurement of pFAK and integrin α5β1 expression on the remodeling vessel was calculated as % of corresponding fluorescent positive length along the $CD31^+$ EC area. Fibronectin deposition was calculated as fibronectin+ area divided by CD31+ EC area in 56.3 mm^2 area. For comparison of staining

intensities, the values were normalized by the background signals in nonvascularized regions.

Immunoblotting

WT mice were anesthetized with an intraperitoneal injection of ketamine (100 mg/kg) and xylazine (12.5 mg/kg) at 3 days after MI or Sham procedure. Hearts were harvested, washed in ice-cold PBS, and the infarct region was excised. The protein concentration of the supernatants was quantitated using the detergent-insensitive Pierce BCA protein assay kit (Thermo Scientific, 23227). Lamni buffer was added to total protein lysates and samples were denatured at 95°C for 5 min. Aliquots of each protein lysate (50 µg) were subjected to SDS–polyacrylamide gel electrophoresis. After electrophoresis, proteins were transferred to nitrocellulose membranes and blocked for 30 min with 5% skim milk in TBST (0.1% Tween 20 in TBS). After blocking, the membranes were incubated with the following primary antibodies at 4°C overnight: anti-FOXO1 (rabbit monoclonal, Cell Signaling, 2880); anti-Angpt1 (goat polyclonal, R&D, AF923); anti-Angpt2 (rat monoclonal, R&D, mab7186); anti-Tie1 (rabbit polyclonal, Abcam, ab111517); anti-heparanase (rabbit polyclonal, Prospec, Ins-26-2); anti-GAPDH (rabbit monoclonal, Cell Signaling, #5174). After washes, membranes were incubated with anti-rabbit (Cell Signaling, #7074), anti-rat (Cell Signaling, #7077), or anti-goat (Jackson ImmunoResearch, 705-035-003) secondary peroxidase-coupled antibody for 1 hr at RT. Target proteins were detected using ECL western blot detection solution (Millipore, WBKLS0500).

Immunoprecipitation

To examine Tie2 phosphorylation in the hearts of adult WT or *Angpt2*$^{i\Delta EC}$ mice that were subjected to MI or Sham procedure, and they were harvested at 3 days after the procedure. Infarct region was excised and homogenized in ice-cold RIPA buffer containing protease and phosphatase inhibitors (Roche). The lysates were centrifuged at 14,000 g at 4°C for 15 min and 5 mg of supernatants were used for immunoprecipitation using anti-Tie2 antibody (R&D systems, AF762). The immunoprecipitates were incubated with 20 µl of pre-washed protein A agarose beads (GE Healthcare) for 2 hr. Beads with immunoprecipitates were washed 3 times with cold lysis buffer, heated in NuPAGE sample buffer (Invitrogen) at 95°C for 5 min, subjected to SDS-PAGE on 4–12% NuPAGE Bis-Tris gels (Invitrogen), transferred to nitrocellulose membrane (Invitrogen), and probed with horseradish peroxidase (HRP)-conjugated anti-phosphotyrosine 4G10 antibody (Millipore). The blots were developed using ECL western blot detection solution (Millipore, WBKLS0500). The membranes were stripped and re-probed with anti-Tie2 (Santa Cruz, Sc-324) antibody.

***In vivo* permeability assay**

At 3 days after MI, 200 µl of FITC-conjugated dextran (5 mg/ml, 40 kDa, Sigma-Aldrich) was intravenously injected 30 minutes prior to sacrifice, and the hearts were snap frozen, sectioned, and stained for visualization of dextran and ECs by immunofluorescence staining. Vascular leakage was quantified as FITC-dextran$^+$ area divided by total CD31$^+$ EC areas in three random 0.017 mm^2 areas of each infarct border and averaged.

Lectin perfusion assay

For lectin perfusion assay, 100 µl of DyLight® 488-conjugated tomato-lectin (1 mg/ml, Vector laboratory, DL-1174) was intravenously injected into the mice. At 15 minutes afterward, the mice were anesthetized and perfused by intra-cardiac injection of PBS to remove intravascular lectin. Vascular perfusion area was calculated as the percentage of FITC-Lectin$^+$ area divided by CD31$^+$ EC area in three random 0.017 mm^2 fields of each infarct border and averaged.

Assays for urine albumin and creatinine

Urine albumin and creatinine concentrations were measured by ELISA using commercial kits (Abcam, ab108792 for albumin; ab65340 for creatinine). Mouse urine samples were freshly obtained from 8- to 10-week-old male WT or *Angpt2*$^{i\Delta EC}$ mice, diluted 100-fold for albumin or 10-fold for creatinine, and measured according to the manufacturer's instruction using a Spectra MAX340 plate reader (Molecular Devices).

RNA extraction, cDNA synthesis, and quantitative RT-PCR

Total RNA was extracted from samples using Trizol (Invitrogen, #15596018) according the manufacturer's protocols. A total of 1 µg of extracted RNA was transcribed into cDNA using GoScript™ Reverse Transcription System (Promega, A5004). cDNA was mixed with primers and FastStart SYBR Green Master (Roche, #04913914001), and mRNA expression levels were measured by qRT–PCR on CFX Connect Real-Time PCR Detection System (Bio-Rad, #1855201). The primers were designed using Primer-BLAST and the list of qRT-PCR primers

used in this study is described in Supplemental Table 1. Primer reaction specificity was confirmed by melting curve analysis. Relative gene expression was analyzed by ΔΔCt method using the CFX Manager software (Bio-Rad, ver. 3.0).

Integrin α5β1 inhibitory peptide treatment

The integrin α5β1 inhibitory peptide (ATN-161) was purchased from R&D systems (Cat # 6058/10). ATN-161 (30 mg/kg) or vehicle was injected intraperitoneally a total of 3 times every 2 days into WT mice subjected to MI surgery.

Cell culture

Human Umbilical Vein Endothelial Cells (HUVECs) were cultured according to the manufacturer's protocols (Lonza, Walkersville, Maryland). In brief, the cells were cultured in endothelial growth medium (EGM2, Lonza) in culture dishes coated with 0.1% gelatin and incubated in a humidified atmosphere with 5% $CO2$ at 37°C. The cells used were between passages 3 to 6. To facilitate the binding affinity of integrin receptors, HUVECs were preincubated with 1mM $Mn2^{+}$ for 30 minutes before each experiment. To induce FAK phosphorylation, HUVECs were stimulated with PBS or recombinant human Angpt2 (R&D #623-AN-025, 10 ng/ml) for 30 minutes.

RNA interference

HUVECs were transfected with a pool of siRNAs using Lipofectamine® RNAiMAX (Invitrogen) according to the manufacturer's protocols. The following target sequences were used:

human *ITGA5* (Cat #3678, Bioneer, Daejeon, Korea) and *ITGB1* (5'-TGATAGATCCAATGGCTTA-3'). *GL2* siRNA (5'- CGTACGCGGAATACTTCGA -3') were used as negative control.

Isolation of endothelial cells from the infarcted heart

WT or *Angpt2*$^{i\Delta/\Delta}$ mice were anesthetized with an intraperitoneal injection of ketamine (100 mg/kg) and xylazine (12.5 mg/kg) at 12-14 days after MI. Hearts were quickly dissected and washed in ice-cold PBS. After clearance of blood, infarct region was excised, minced with fine scissor into small pieces and digested in a DMEM buffer containing collagenase type 2 (2 mg/ml, Worthington, LS004177), Dispase (1 mg/ml, Gibco, 17105-041), and DNase I (0.1 mg/ml, Roche, 10104159001) at 37°C for 20 minutes. Tissues were gently agitated, strained with a 40-µm nylon mesh to remove cell clumps, and incubated with ACK lysis buffer for 2 minutes to remove erythrocytes. Leukocytes were depleted from the isolated cells by use of anti-mouse CD45 microbeads and AutoMACS separation system (Miltenyi Biotec), and the remaining cells were stained with PE-conjugated rat anti-CD31 (BD Pharmingen, 553373). The mouse lineage antibody cocktail (BD Pharmingen, 51-9006964) specific to CD3e chain, CD11b, CD45R/B220, TER119, Ly-6G, and Ly-6C was used to exclude contamination by hematopoietic lineage. To discriminate dead cells, cells were stained with DAPI (Sigma-Aldrich) and cell sorting was performed with FACSAria II (BD Biosciences).

RNA isolation, library preparation and RNA sequencing

For RNA isolation, freshly isolated ECs from infarcted heart by FACS sorting were used. Total RNA was isolated using Trizol reagent (Invitrogen). RNA quality was assessed by Agilent 2100

bioanalyzer using the RNA 6000 Nano Chip (Agilent Technologies), and RNA quantification was performed using ND-2000 Spectrophotometer (Thermo Inc.). For library preparation, SENSE 3’ mRNA-Seq Library Prep Kit (Lexogen, Inc.) were used according to the manufacturer’s instructions. In brief, each 500ng total RNA were prepared and an oligo-dT primer containing an Illumina-compatible sequence at its 5’ end was hybridized to the RNA and reverse transcription was performed. After degradation of the RNA template, second strand synthesis was initiated by a random primer containing an Illumina-compatible linker sequence at its 5’ end. The double-stranded library was purified by using magnetic beads to remove all reaction components. The library was amplified to add the complete adapter sequences required for cluster generation. The finished library is purified from PCR components. High-throughput sequencing was performed as single-end 75 sequencing using NextSeq 500 (Illumina, Inc.). For RNA sequencing (RNA-Seq), SENSE 3’ mRNA-Seq reads were aligned using Bowtie2 version 2.1.0 (3). Bowtie2 indices were either generated from genome assembly sequence or the representative transcript sequences for aligning to the genome and transcriptome. The alignment file was used for assembling transcripts, estimating their abundances and detecting differential expression of genes. Differentially expressed gene (DEG) were determined based on counts from unique and multiple alignments using EdgeR within R version 3.2.2 (R development Core Team) using BIOCONDUCTOR version 3.0 (4). The RT (Read Count) data were processed based on Quantile normalization method using the Genowiz™ version 4.0.5.6 (Ocimum Biosolutions). All gene expression values from RNA-Seq were changed to $\log_2$ values and analyzed further. Gene set enrichment analysis (GSEA) was performed with version 5.2 of the Molecular

Signatures Database (http://www.broadinstitute.org/gsea/msigdb), and the gene sets that had less than 0.05 nominal P values were stated. The Original data are available in the National Center for Biotechnology Information's Gene Expression Omnibus (accession number GSE 105223).

Echocardiography

Transthoracic echocardiography studies were performed at 3 weeks after MI procedure under anesthesia with inhalation of isoflurane (1.5%-3.0%) using a nose cone. Mice were placed on a warm board in a supine position, and warm acoustic gel was applied to the scan field as a coupling medium, and images were acquired using a commercial high-frequency (40 MHz) ultrasound system (Prospect, S-Sharp Corporation, New Taipei City, Taiwan). To evaluate the cardiac function, two-dimensionally targeted M-mode images at a level of papillary muscle and 2D B-mode cine loops of parasternal short- and long-axis view of the LV were acquired. A single observer blinded to the experimental conditions as well as the genotypes performed all image analyses.

Supplementary Table 1. List of Primer Sets for Quantitative Real-Time RT-PCR for mouse samples

Gene	Primer sequence (5' - 3')	
Mouse *Angpt2*	Forward	ATACAAAGAGGGCTTCGGGAG
	Reverse	TTGCCTTCCCAGTCCTTCAG
Mouse *Tie2*	Forward	GGAAGAACGGAAGACATACGTG
	Reverse	CTAGGCTGCTTCTTCCGCAG
Mouse *Lamc2*	Forward	GCATCCTACACCTCATAGACCAG
	Reverse	CAGACATCAAGGGCCGAAG
Mouse *Col12a1*	Forward	CCCTGGTGAACGAGGTTTG
	Reverse	GGGCCTCTTGAACCTGTAGAC
Mouse *Wnt5a*	Forward	GAAGCAGGCCGTAGGACAG
	Reverse	CAGCACGTCTTGAGGCTACAG
Mouse *Pcolce*	Forward	AAGCAGTACAAGCGGTCAGG
	Reverse	TCACTGTTCCTGTCACCACTAGG
Mouse *Fstl3*	Forward	GCTGTCAAAAGTCTTGCGCTC
	Reverse	TAACGTTGTTGTTGCCACAGAG
Mouse *GAPDH* (housekeeping gene)	Forward	CTGAACGGGAAGCTCACTG
	Reverse	CATACTTGGCAGGTTTCTCCAG

References for Supplemental Methods

1. Yan X, Zhang H, Fan Q, Hu J, Tao R, Chen Q, et al. Dectin-2 deficiency modulates th1 differentiation and improves wound healing after myocardial infarction. *Circulation research.* 2017;120(7):1116-29.

2. Han S, Lee SJ, Kim KE, Lee HS, Oh N, Park I, et al. Amelioration of sepsis by TIE2 activation-induced vascular protection. *Science translational medicine.* 2016;8(335):335ra55.

3. Langmead B, and Salzberg SL. Fast gapped-read alignment with Bowtie 2. *Nature methods.* 2012;9(4):357-9.

4. Gentleman RC, Carey VJ, Bates DM, Bolstad B, Dettling M, Dudoit S, et al. Bioconductor: open software development for computational biology and bioinformatics. *Genome biology.* 2004;5(10):R80.

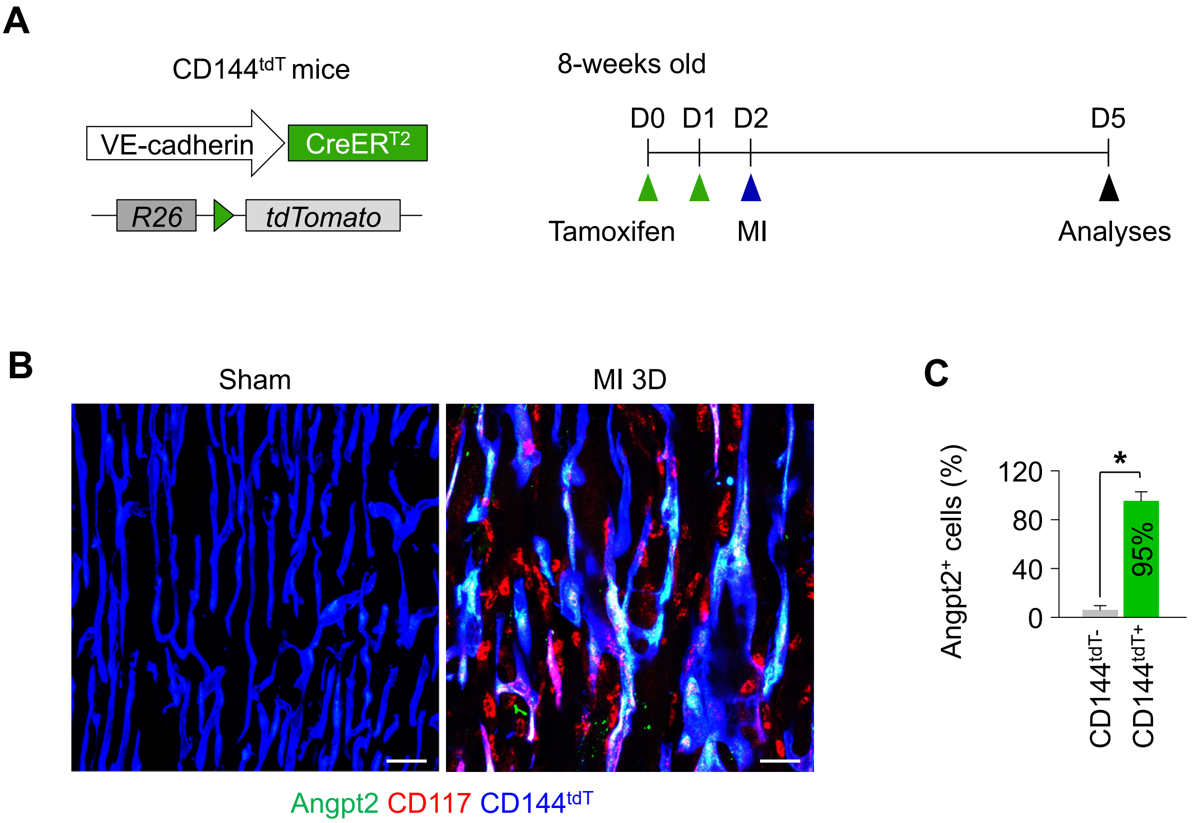

Supplemental Figure 1. Expression of Angp2 is mainly confined to ECs, rather than circulating endothelial cells

Adult VE-Cadherin-Cre-ER^{T2}/*Rosa26-tdToamto* (CD144^{tdT}) mice were subject to MI or Sham (Sh) procedure, hearts were harvested at 3 days after MI, and indicated molecules at the infarct border were detected by immunostaining. (**A**) Diagram depicting preparation of CD144^{tdT} mice and experimental schedule. (**B** and **C**) Images and comparison of Angpt2 expression in cardiac resident- (CD144^{tdT+}) or circulating- (CD117^{+}) ECs. Note that ~95% of Angpt2+ expression was co-localized with CD144^{tdT}. n = 4, each group. Scale bars: 20 μm. *$P < 0.05$ versus Sham by Mann-Whitney *U* test. Error bars represent mean ± s.d.

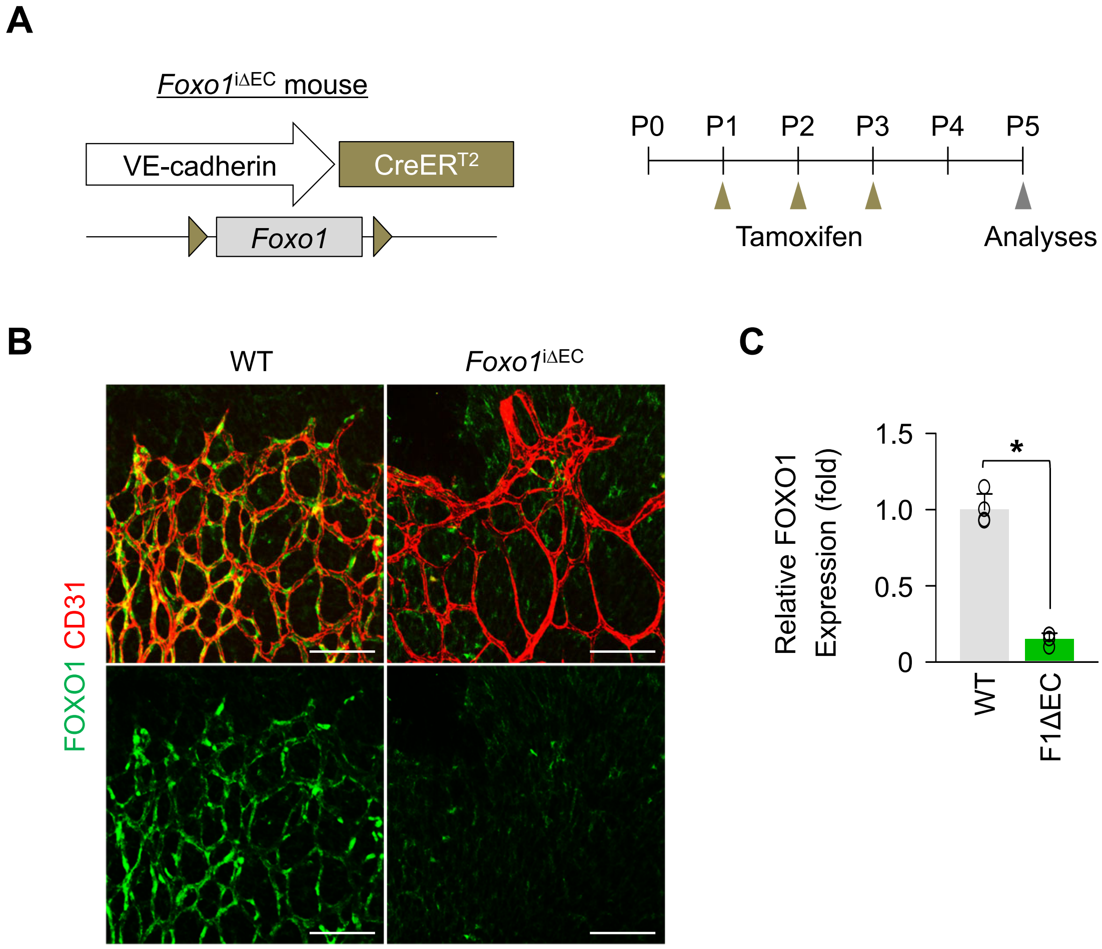

Supplemental Figure 2. Efficacy of EC-specific depletion of *Foxo1*

(**A**) Diagram depicting the experiment schedule for EC-specific depletion of *Foxo1* in retinal vessels from P1 and their analyses at P5 in WT and *Foxo1*$^{i\Delta EC}$ mice. Note the disorganized and enlarged retinal vasculature of *Foxo1*$^{i\Delta EC}$ mice. (**B** and **C**) Images and comparison of FOXO1 in CD31^{+} retinal vessels at P5 in WT and *Foxo1*$^{i\Delta EC}$ mice (n = 3, each group). Scale bars: 100 μm. *P < 0.05 versus WT by Mann-Whitney *U* test. Error bars represent mean ± s.d.

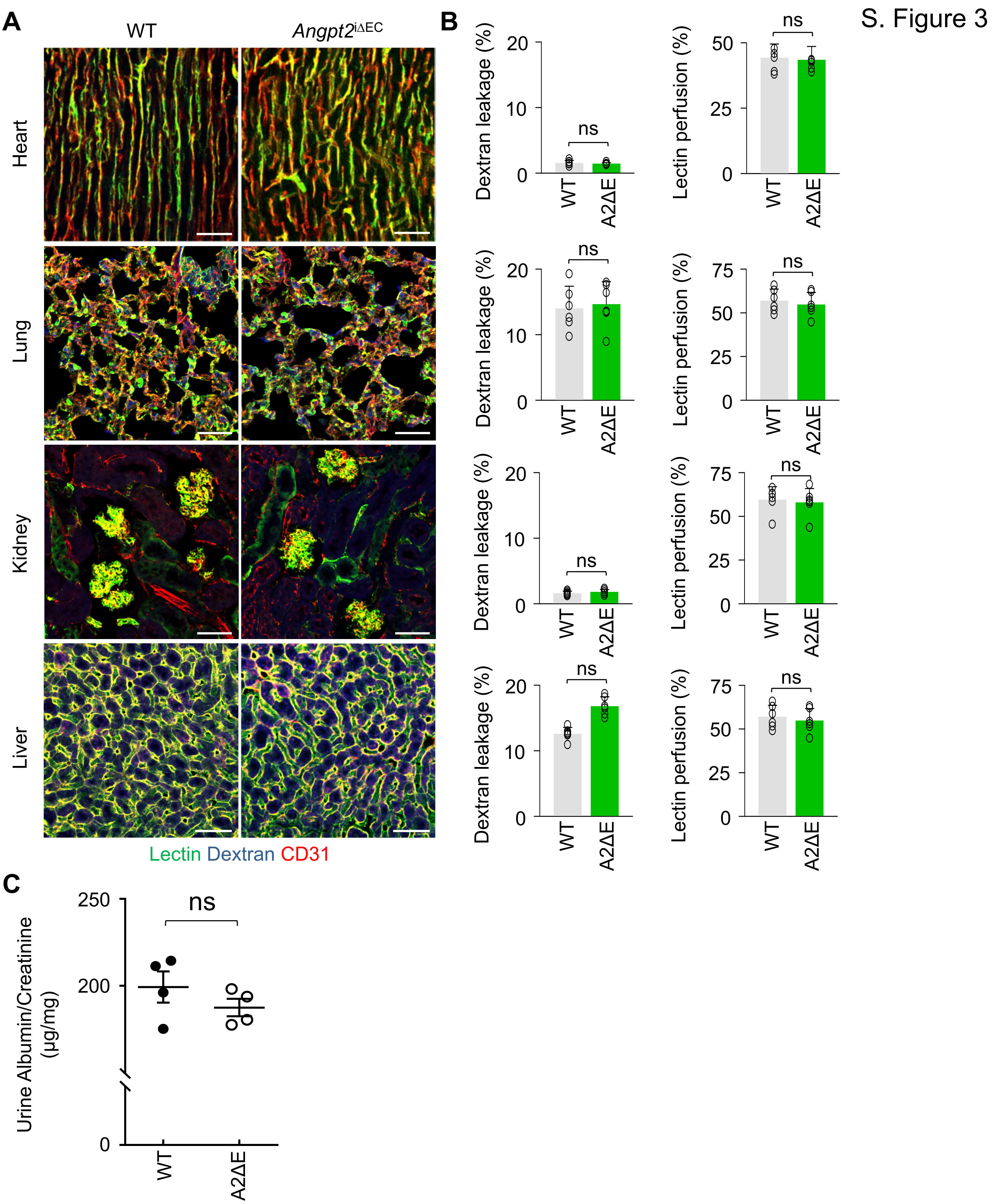

Supplemental Figure 3. EC-specific depletion of *Angpt2* does not alter systemic vascular function and renal function. (**A** and **B**) Heart, lung, kidney and liver was harvested from adult WT or *Angpt2*$^{i\Delta EC}$ (A2ΔE) mice, and further processed for immunostaining. (**A**) Immunofluorescence images for dextran leakage and FITC-lectin perfusion. Scale bars: 50 µm. (**B**) Comparisons of indicated parameters. n = 6, each group. ns, not significant. *P < 0.05 versus WT by Mann-Whitney *U* test. (**C**) Urine albumin-to-creatinine ratio of adult WT and *Angpt2*$^{i\Delta EC}$ (A2ΔE) mice. Note that there was no significant difference in urinary albumin-to-creatinine ratio between the two groups. n = 4, each group. ns, not significant. Error bars represent mean ± s.d.

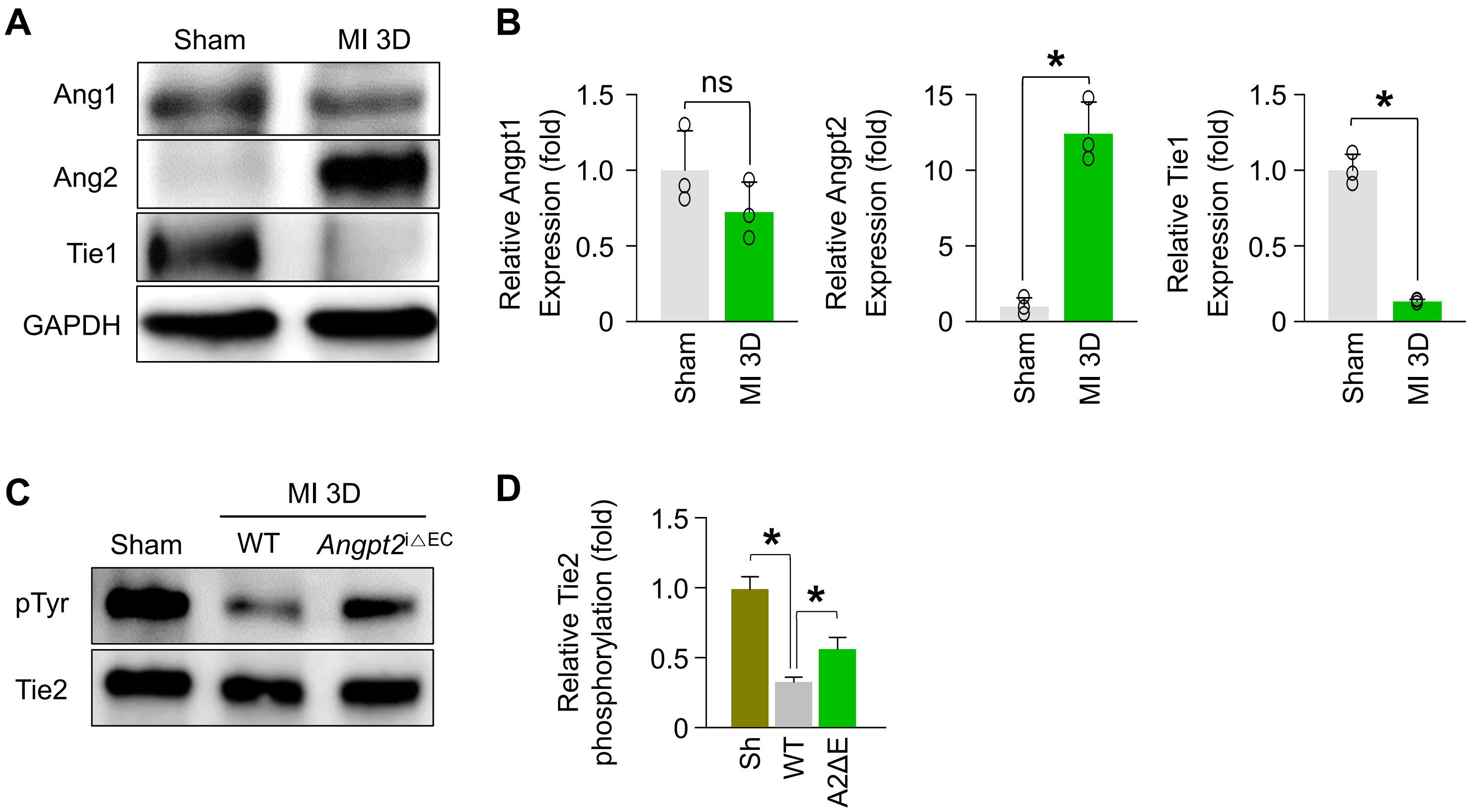

Supplemental Figure 4. Protein level of each Angpt/Tie system molecules at the infarct border after MI
Adult WT or *Angpt2*$^{i\Delta EC}$ (A2ΔE) mice were subject to MI or Sham (Sh) procedure, hearts were harvested at 3 days after MI, and indicated molecules at the infarct border were detected by immunoblotting. (**A** and **B**) Immunoblot and densitometric analyses of indicated proteins at the infarct border after MI. n=3, each group. ns, not significant. *P < 0.05 versus Sham by Mann-Whitney *U* test. (**C**) *In vivo* Tie2 phosphorylation at the infarct border after MI or Sham procedure assessed by immunoprecipitation method. Similar findings were observed in 3 independent experiments. (**D**) Densitometric analyses for relative phosphorylation ratios of Tie2 is shown. *P < 0.025 by Kruskal-Wallis test followed by Mann-Whitney *U* tests for *post hoc* pairwise comparisons. Error bars represent mean ± s.d.

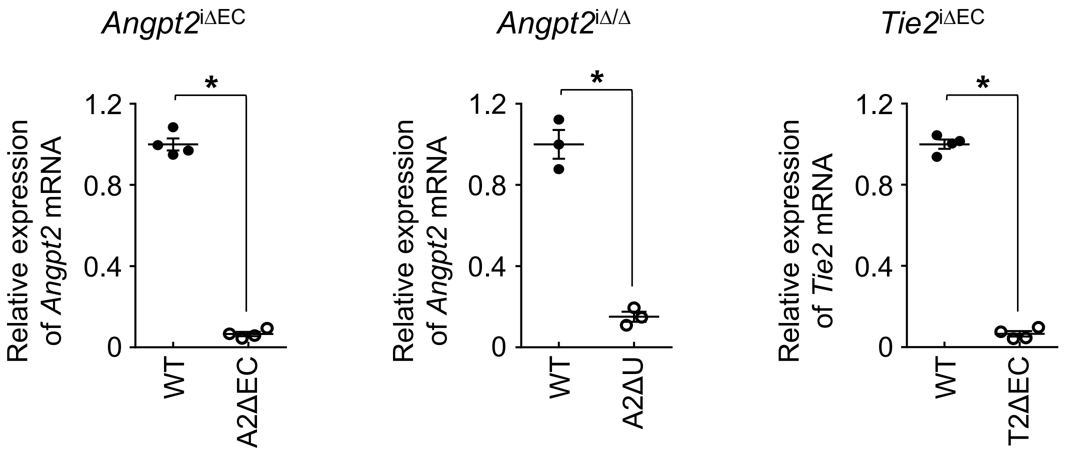

Supplemental Figure 5. Efficacy of gene deletion confirmed by quantitative PCR

The lung ECs of adult WT, *Angpt2*$^{i\Delta EC}$ (A2ΔEC), *Angpt2*$^{i\Delta/\Delta}$ (A2ΔU) or *Tie2*$^{i\Delta EC}$ (T2ΔEC) mice were freshly isolated at 2 weeks after tamoxifen administration. The deletion efficacy of each gene in the ECs was measured by quantitative PCR of each gene (n=3-4, each group). *P < 0.05 versus WT by Mann-Whitney *U* test. Error bars represent mean ± s.d.

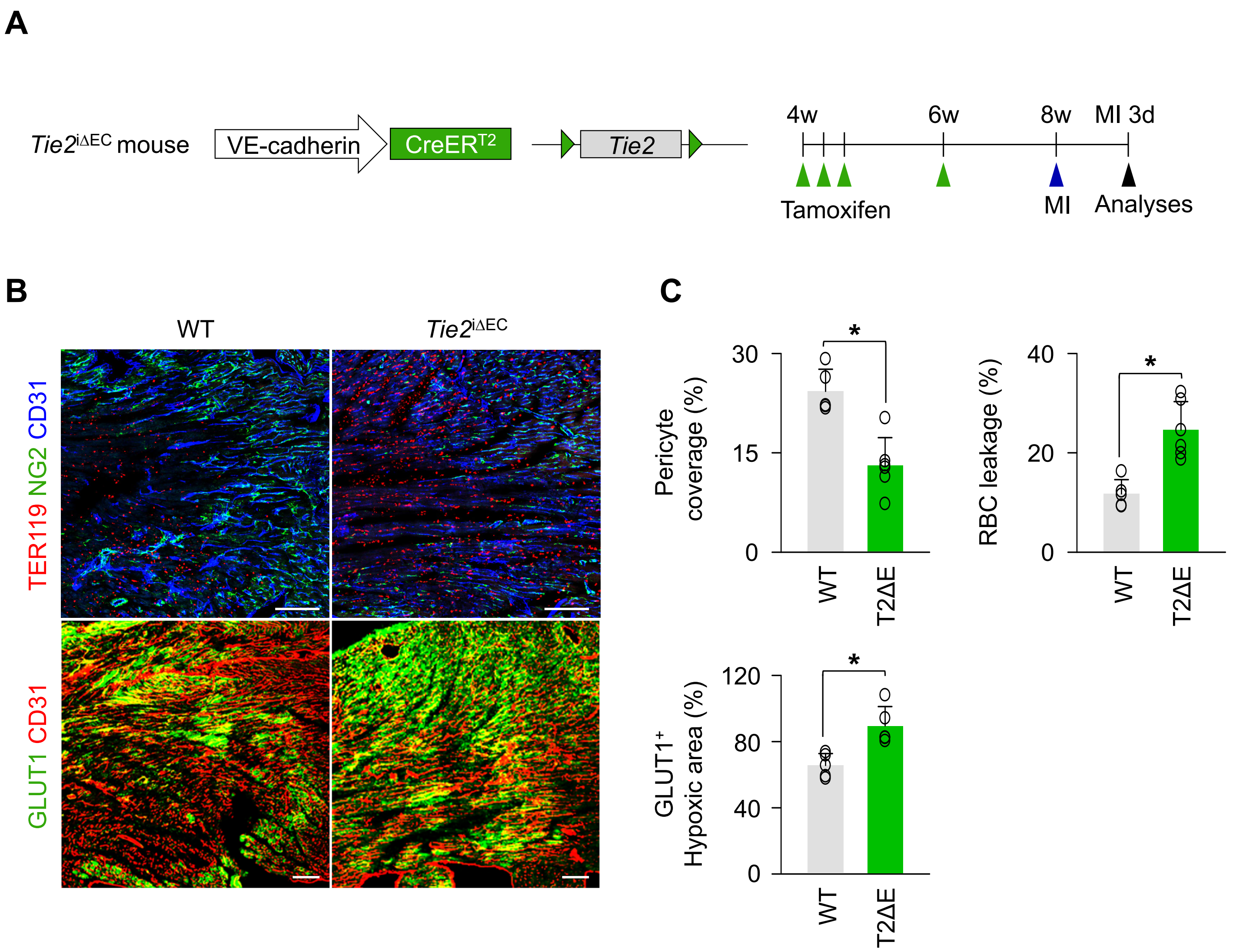

Supplemental Figure 6. Pericyte detachment, vascular leakage, and cardiac hypoxia is more exacerbated by EC-specific depletion of *Tie2*

Adult WT, or *Tie2*$^{i\Delta EC}$ (T2ΔE) mice were subject to MI, hearts were harvested at 3 days after MI, and indicated molecules in heart sections at the infarct border were detected by immunostaining. (**A**) Diagram depicting generation of *Tie2*$^{i\Delta EC}$ mice and their experiment schedule. (**B** and **C**) Images and comparisons of NG2^{+} pericyte coverage, TER119^{+} RBC leakage, and GLUT1^{+} hypoxic area at 3 days after MI. n = 5-6, each group. Scale bars: 100 μm. *$P < 0.05$ versus WT by Mann-Whitney *U* test. Error bars represent mean ± s.d.

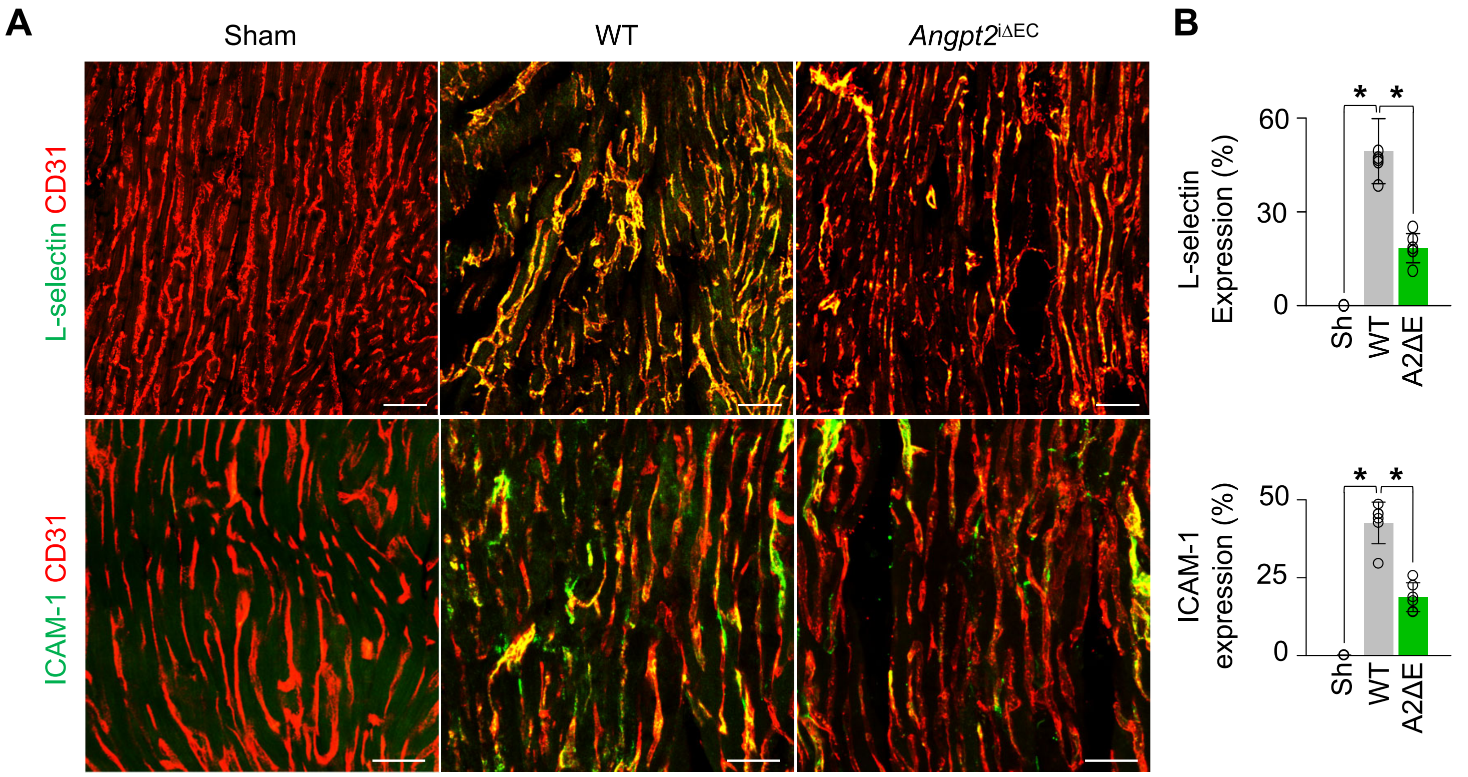

Supplemental Figure 7. EC-specific depletion of *Angpt2* mitigates adhesion molecular expression after MI

Adult WT or *Angpt2*$^{i\Delta EC}$ (A2ΔE) mice were subject to MI or Sham (Sh) procedure, hearts were harvested at 3 days after MI, and indicated molecules in heart sections were detected by immunostaining. (**A** and **B**) Images and comparisons of L-selectin and ICAM-1 expression in the ECs. n = 5-6, each group. Scale bars: 50 μm. *P < 0.025 by Kruskal-Wallis test followed by Mann-Whitney *U* tests for *post hoc* pairwise comparisons. Error bars represent mean ± s.d.

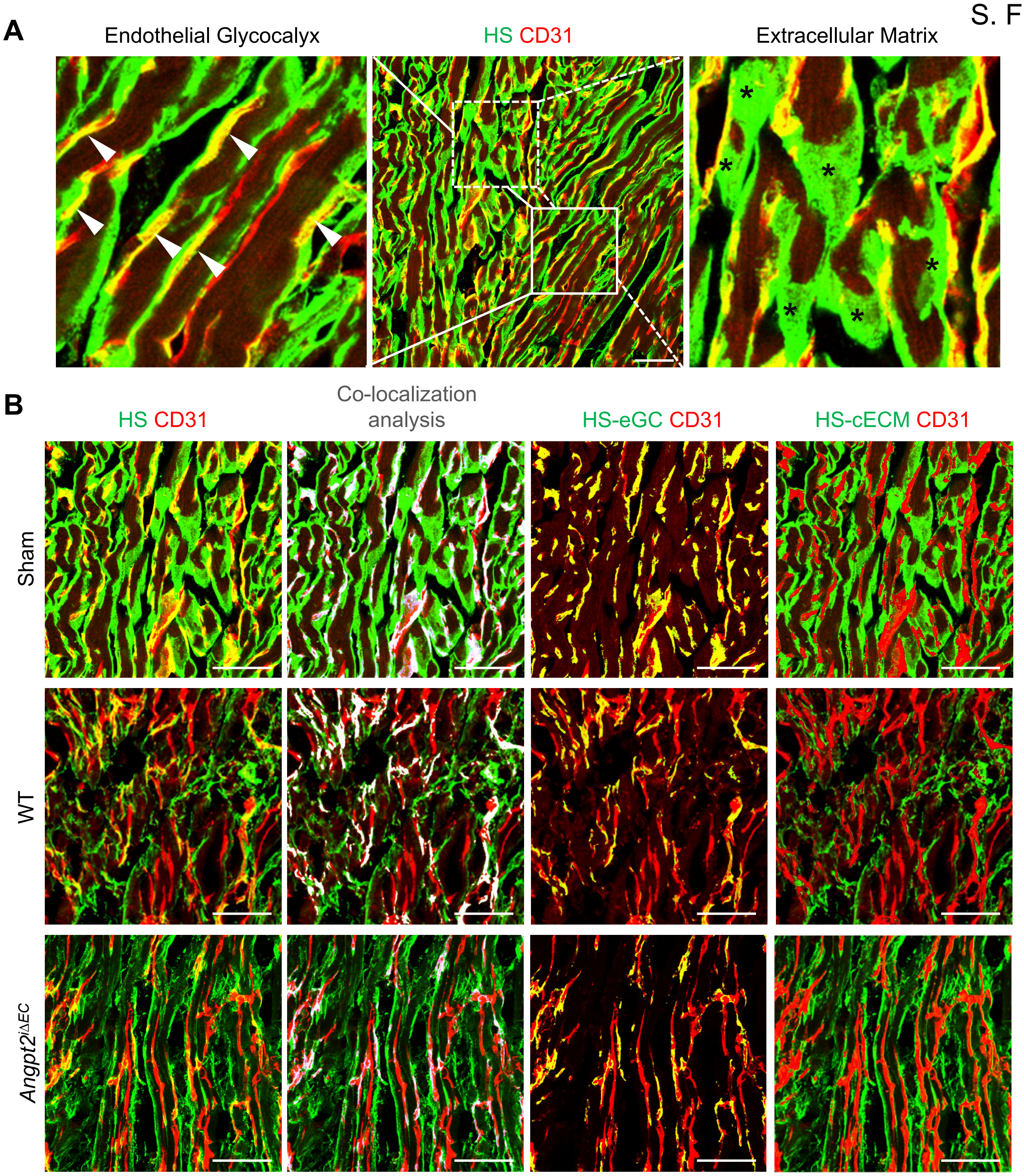

Supplemental Figure 8. Visualization of endothelial glycocalyx and cardiac ECM by immunofluorescence staining for heparan sulfate Adult WT or *Angpt2*$^{i\Delta EC}$ mice underwent MI or sham surgical procedures. Hearts were harvested 3 days post-MI, and the indicated molecules were detected by immunofluorescence staining in heart sections at the infarct border zone. (A) High-magnification and differential fluorescence imaging of Fig. 6E demonstrating heparan sulfate (HS) distribution in sham control myocardium. The left magnified panel reveals thin, linear HS structures coating endothelial cells, representing the endothelial glycocalyx (eGC, white arrowheads). The right magnified panel shows thicker HS deposits surrounding cardiomyocytes, corresponding to the cardiac extracellular matrix (cECM, black asterisks). Scale bar, 50 μm. Original image derived from the sham condition in Fig. 6E. (B) High-magnification and differential fluorescence imaging of Fig. 6E illustrating eGC and cECM degradation at 3 days post-MI in both WT and *Angpt2*$^{i\Delta EC}$ groups. The middle panels display colocalization of CD31 and HS signals (white), operationally defined as eGC (HS-eGC). The remaining HS signal, excluding the eGC fraction, was designated as cardiac ECM (HS-cECM) as quantified in Fig. 6E. Colocalization analysis and image segmentation were performed using ImageJ software. Scale bars, 50 μm.

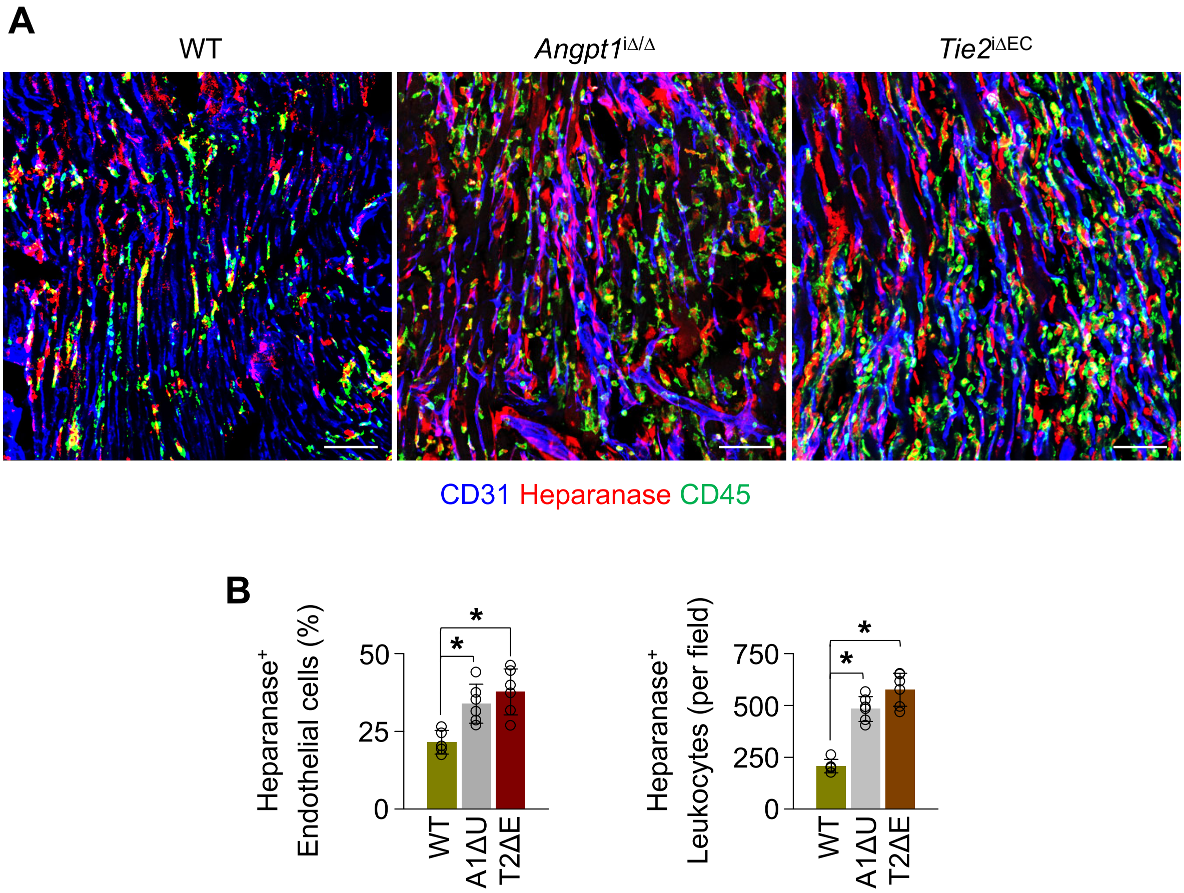

Supplemental Figure 9. Angpt1-Tie2 signaling suppresses heparanase expression in ECs and recruitment of heparanase expressing leukocytes at the border zone

Adult WT, *Angpt1*$^{i\Delta/\Delta}$ (A1ΔU), or *Tie2* $^{i\Delta EC}$ (T2ΔE) mice were subject to MI procedures, hearts were harvested at 3 days after MI, and indicated molecules in heart sections at the infarct border were detected by immunostaining. (**A** and **B**) Images and comparisons of heparanase expression in ECs and infiltrating leukocytes. n = 5-6, each group. Scale bars: 100 μm. *P < 0.025 by Kruskal-Wallis test followed by Mann-Whitney *U* tests for *post hoc* pairwise comparisons. Error bars represent mean ± s.d.

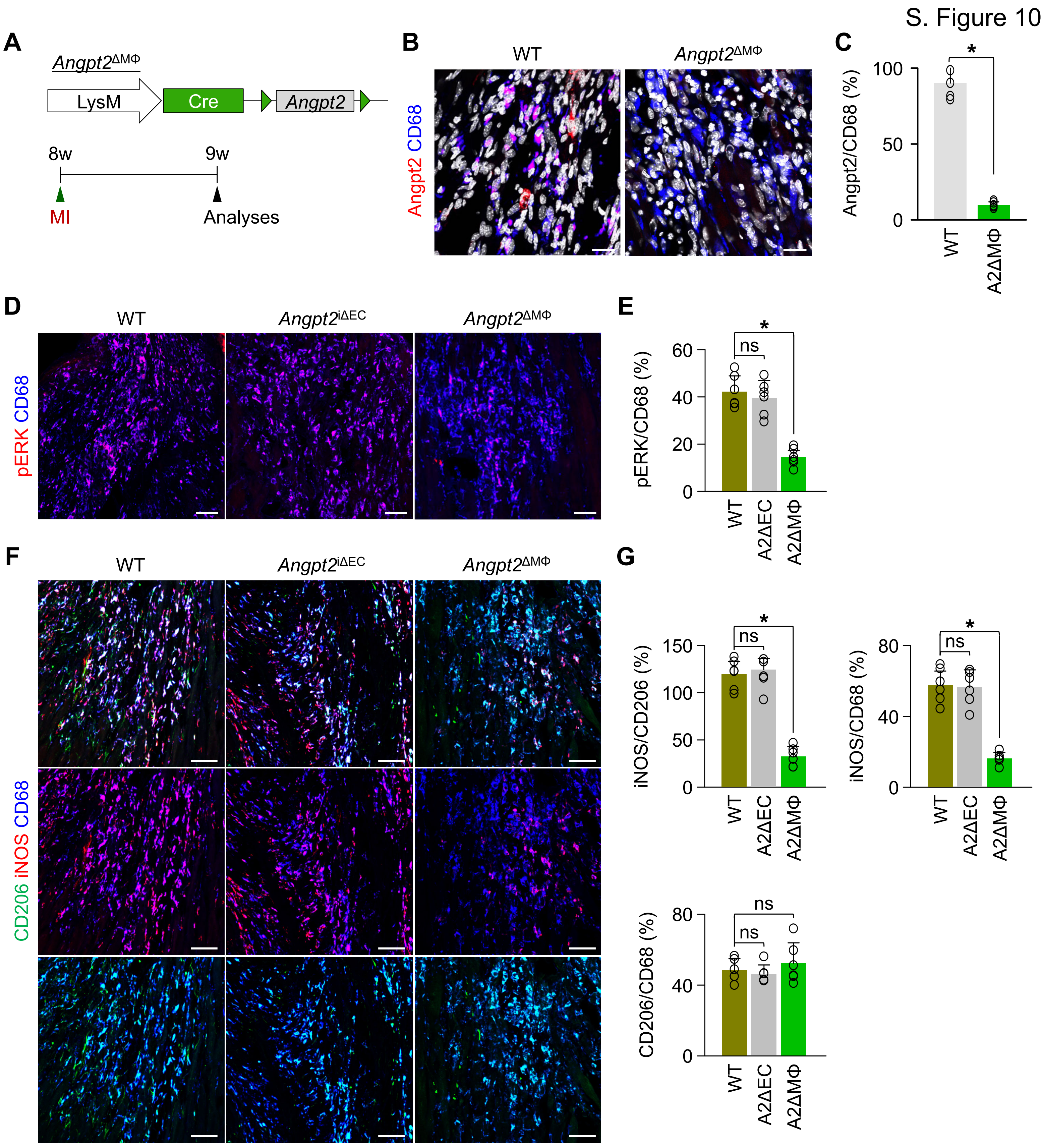

Supplemental Figure 10. Macrophage-derived Angpt2 plays a dominant role in ERK phosphorylation and proinflammatory polarization Adult WT, *Angpt2*$^{i\Delta EC}$ (A2ΔEC) or *Angpt2*$^{\Delta M\Phi}$ (A2ΔMΦ) mice were subject to MI procedure, hearts were harvested at 7 days after MI, and indicated molecules in heart sections at the infarct border were detected by immunostaining. MΦ, macrophage. (**A**) Diagram depicting preparation of animals and experimental schedules. (**B** and **C**) Images and comparisons of Angpt2 expression on macrophages. n = 5, each group. Scale bars: 20 μm. (**D** and **E**) Images and comparisons of pERK in CD68$^+$ macrophages. n = 5-6, each group. Scale bars: 50 μm. (**F**) Images of expression of iNOS and CD206 in CD68$^+$ macrophages. Scale bars: 50 μm. (**G**) Comparisons of indicated parameters. n = 5, each group. *P < 0.05 by Mann-Whitney *U* test. ns, not significant. Error bars represent mean ± s.d.

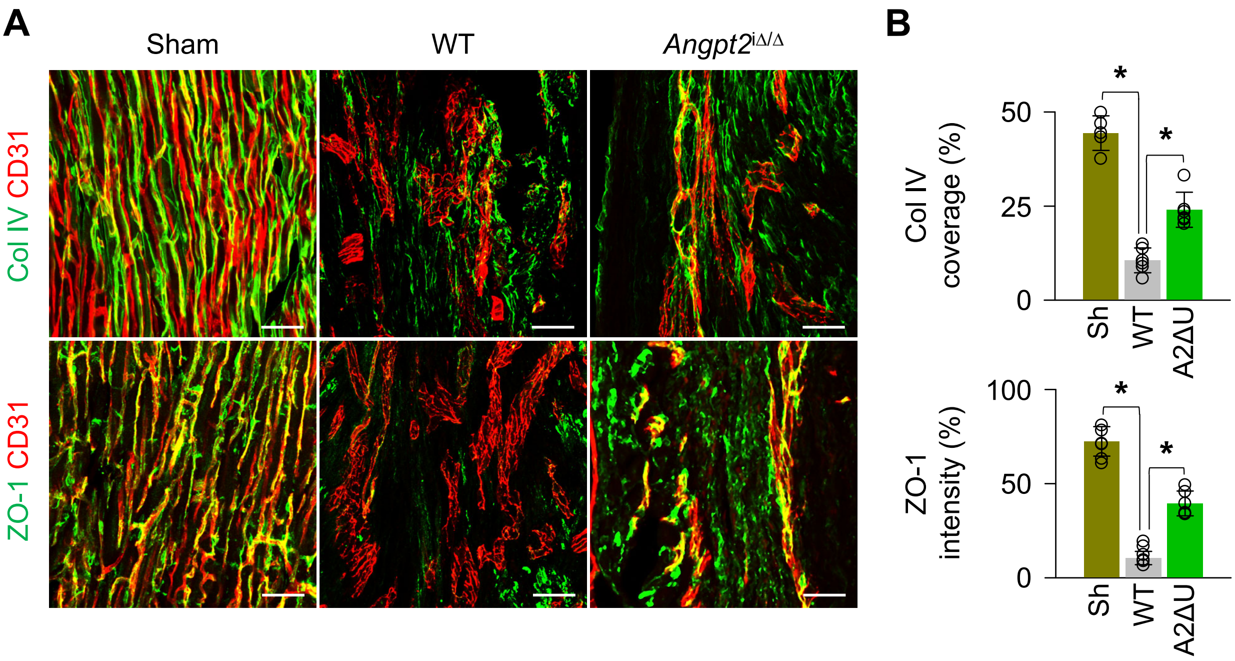

Supplemental Figure 11. Genetic depletion of *Angpt2* mitigates adverse vascular remodeling

Adult WT or *Angpt2*$^{i\Delta/\Delta}$ (A2ΔE) mice were subject to MI or Sham (Sh) procedure, hearts were harvested at 2 weeks after MI, and indicated molecules in heart sections at the infarct border were detected by immunostaining. (**A** and **B**) Images and comparisons of distributions of Col IV^{+} basement membrane and ZO-1^{+} tight junction in infarct border zone ECs. n = 5-6, each group. Scale bars: 50 μm. *$P < 0.025$ by Kruskal-Wallis test followed by Mann-Whitney *U* tests for *post hoc* pairwise comparisons. Significance was adjusted for multiple comparisons using Bonferroni's method. Error bars represent mean ± s.d.

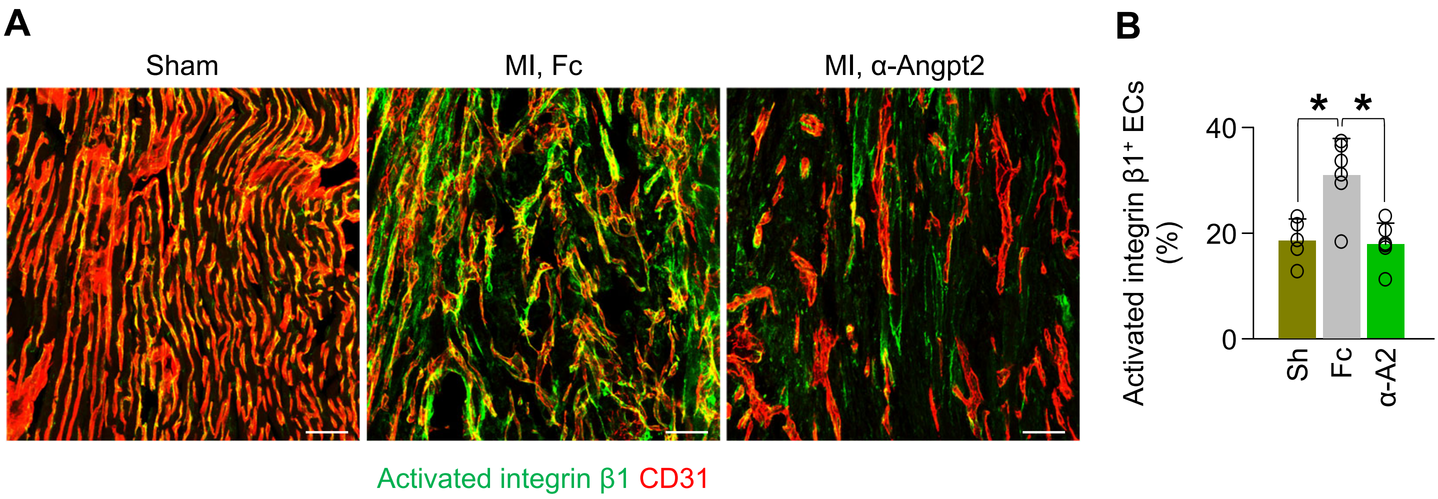

Supplemental Figure 12. Antibody-mediated neutralization of Angpt2 suppresses excessive integrin β1 activation in the infarct border ECs

Adult WT mice were subject to MI or Sham (Sh) procedure, then Fc or α-Angpt2 (α-A2) (20 mg/kg, intraperitoneally) was administered to WT or *Angpt2*$^{i\Delta/\Delta}$ mice at 6 hours after MI. Hearts were harvested at 7 days after MI, and indicated molecules in heart sections were detected by immunostaining. (**A** and **B**) Images and comparison of Activated integrin β1 (CD29) expression in the infarct border ECs. Scale bars: 50 μm. n = 5-6, each group. *P < 0.05 by Mann-Whitney *U* test. Error bars represent mean ± s.d.

S. Figure 13

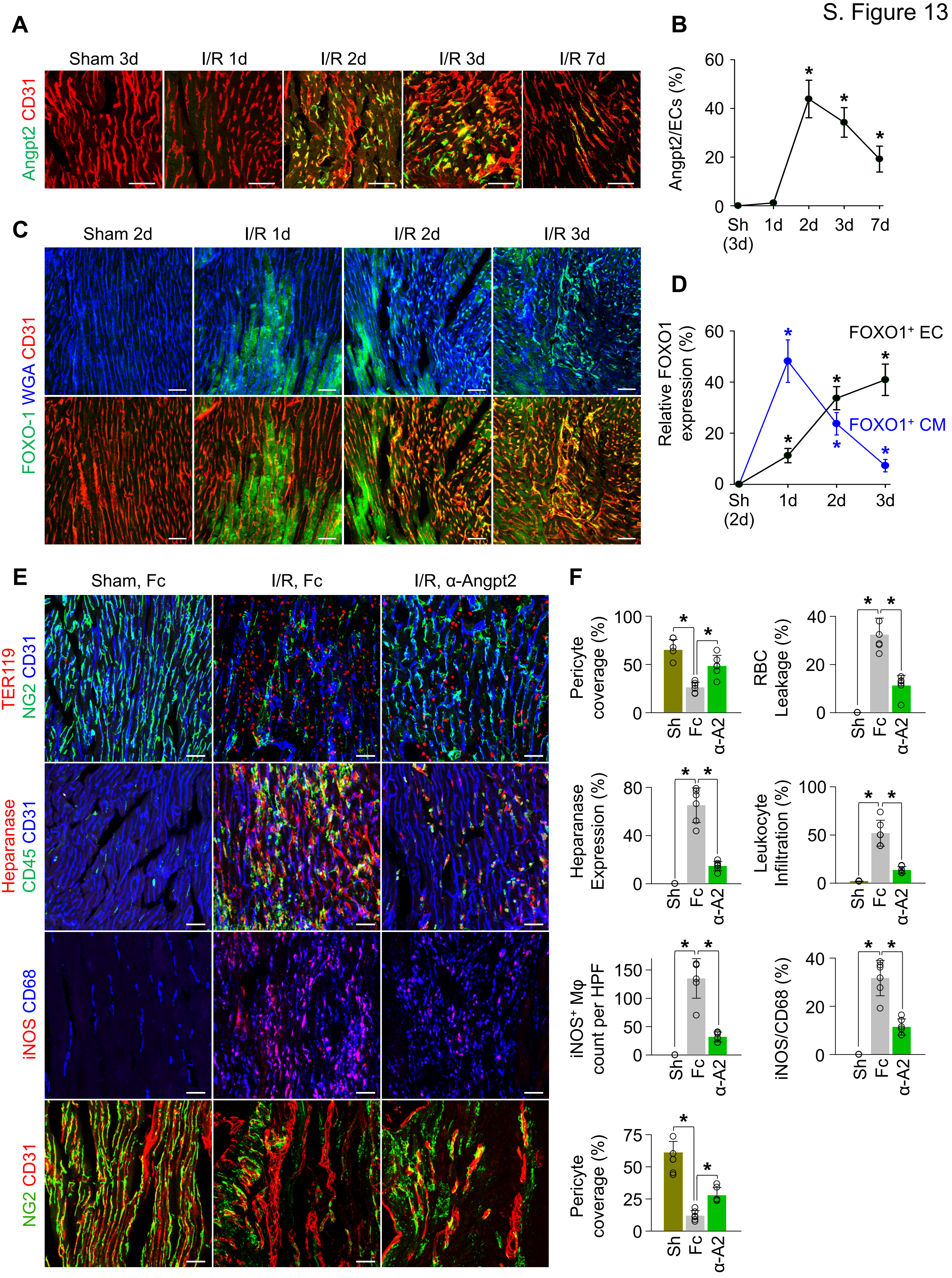

A
Sham 3d
I/R 1d
I/R 2d
I/R 3d
I/R 7d
Angpt2 CD31
B
Angpt2/ECs (%)
Sh (3d)
1d
2d
3d
7d
C
Sham 2d
I/R 1d
I/R 2d
I/R 3d
FOXO-1 WGA CD31
D
Relative FOXO1 expression (%)
FOXO1+ EC
FOXO1+ CM
Sh (2d)
E
Sham, Fc
I/R, Fc
I/R, α-Angpt2
TER119 NG2 CD31
Heparanase CD45 CD31
iNOS CD68
NG2 CD31
F
Pericyte coverage (%)
RBC Leakage (%)
Heparanase Expression (%)
Leukocyte Infiltration (%)
iNOS+ Mφ count per HPF
iNOS/CD68 (%)
Sh
Fc
α-A2

Supplemental Figure 13. Angpt2 possesses a crucial role in mediating ischemia-reperfusion injury of heart Adult WT mice were subject to I/R or Sham procedure, hearts were harvested at the indicated days after the procedure, and indicated molecules in heart sections were detected by immunostaining. (**A** and **B**) Temporal changes of Angpt2 expression after I/R in the reperfused area ECs. n=5-6, each time point. Scale bars: 50 μm. (**C** and **D**) Temporal changes of expression and distribution of FOXO1 after I/R. n=5-6, each time point. Scale bars: 50 μm. (**B** and **D**) *P < 0.05 versus Sham by Mann-Whitney *U* test. (**E** and **F**) Fc or α-Angpt2 (α -A2) (20 mg/kg, intraperitoneally) was administered to adult WT mice at 6 hours after I/R procedure, and followed by repeated injection of same dose at 1 week interval. Images and comparisons of $NG2^+$ pericyte coverage, $TER119^+$ RBC leakage, heparanase expression, $CD45^+$ leukocytes infiltration at 3 days after, $iNOS^+$ macrophage count and proportion at 7 days after, and $NG2^+$ pericyte coverage at 14 days after I/R procedure. n = 5-6, each group. Scale bars: 50 μm. Mφ, macrophage. HPF, high power filed. *P < 0.025 by Kruskal-Wallis test followed by Mann-Whitney *U* tests for post hoc pairwise comparisons. Significance was adjusted for multiple comparisons using Bonferroni's method. Error bars represent mean ± s.d.
